# 

**Published:** 1873-07

**Authors:** 


### ARTICLE III.

### *American Dental Convention.*

New York, June 20th, 1873.

*Mr. Editor:*—The committee of arrangements would respectfully inform the members of the profession, that the nineteenth annual meeting of the American Dental Convention will be held at Saratoga Springs, on the 12th day of August, (second Tuesday,) commencing at 10 A. M.

Messrs. Breslin, Gardner & Co., of the Grand Union hotel have generously tendered their large hall, (rooms for committees, &c.,) also, their splendid parlour grounds and band of music for our social entertainment. The town authorities have also offered us a very suitable room for our meeting, in the new and magnificent hall recently erected there. The committee have made arrangements for the display and exhibition of dental materials and appliances of all kinds; also, for clinical operations during the entire session.

Dentists and others desirous of exhibiting instruments, materials, or improvements in any department of dentistry, are particularly invited to present them as early in the session as possible, and to notify the committee of their intention to do so. As August is a very busy month with hotel keepers, it will be advisable for those intending to be present, to notify the committee of their wishes, &c. The committee will see that good; accommodations are provided for all who give timely notice, and for others as far as possible, and will endeavor to make this meeting a pleasant and profitable gathering to all. The profession generally, are cordially invited to be present.

J. G. AMBLER, Chairman of Committee,
*25 West 23rd Street, New York City.*

---

### *American Dental Association.*

The thirteenth annual session of the American Dental Association will be held at Put-in-Bay, an Island in lake Erie, a short distance from Sandusky, Ohio, commencing Tuesday, August 15th, at 10 A. M.

Route—Steamboats leave Detroit daily at 9 A. M.; boats also leave Toledo daily (and Cleveland, I believe) for Put-in-Bay. Passengers either from the West or East, on the Lake Shore and Michigan Southern Railroad, or on any of the roads terminating or passing through Sandusky, can take boats for the island at 9 A. M., and at 3 and 7.30 P. M. Passengers on the Baltimore and Ohio Road arrive at Sandusky at 6.30 P. M., in time for the 7.30 boat.

M. S. Dean, Secretary.

### *The Southern Dental Association.*

The fifth annual meeting of this association will be held in Baltimore, commencing on the last Tuesday in July next. This promises to be one of the most interesting meetings ever held, and we trust it will be well attended by the Southern members of the profession. Efforts are now being made to obtain reduced fare on all railroads in connection with the Baltimore and Ohio, due notice of which will be given.

*On Strictures of the Urethra.*—Results of operations with the dilating urethrotome, with cases. By F. N. Otis, M. D. Publishers, D. Appleton & Co., New York, 1873.

---

### MONTHLY SUMMARY.

### TO READERS AND CORRESPONDENTS.

Communications intended for publication, and exchange journals and papers, should be addressed to the EDITOR OF THE AMERICAN JOURNAL OF DENTAL SCIENCE, 259 N. Eutaw St., Baltimore, Md. All letters relating to the business of the journal, or containing cash remittances, to be directed to Messrs. SNOWDEN & COWMAN. Articles for publication should be received by the editor at least three weeks previous to the first day of the month on which the number in which it is designed for them to appear, is issued.

### CONTENTS:

### ORIGINAL COMMUNICATIONS.

☞ The American Journal of Dental Science will contain fifty pages of reading matter in each number, making a volume of not less than

*Six Hundred* pages

EXCLUSIVE OF ADVERTISEMENTS.

### THE

### MERICAN JOURNAL

### OF

### DENTAL SCIENCE.

F. J. S. GORGAS, M. D., D. D. S., Editor.

e *Journal* is issued on the first of each month and contains not less *fifty* pages of reading matter in each number, exclusive of adver- ents, making a volume of *six hundred* pages per annum.

each number will be found Original Communications, Corres- ence, Selected Articles, Monthly Summary, Bibliographical No- and Editorial Matter, and every effort will be exerted to render *ournal* worthy of the patronage of the Dental profession.

generous co-operation is respectfully solicited from the members e profession; and as the *Journal* has now a printing office of its which materially lessens the cost of its publication, it will be d at the reduced subscription price of

**$2.50 Per Annum in Advance.**

ommunications are respectfully solicited on all subjects pertain- to the practice of dentistry, as well as reports of cases occurring ental practice.

### RATES OF ADVERTISING.

| | 1 MO. | 2 MO. | 3 MO. | 6 MO. | 12 MO. |
|---|---|---|---|---|---|
| PAGE. | $15 00 | $22 50 | $30 00 | $50 00 | $100 00 |
| " | 10 00 | 15 00 | 20 00 | 30 00 | 50 00 |
| " | 7 00 | 11 00 | 15 00 | 20 00 | 30 00 |
| " | 5 00 | 8 00 | 11 00 | 15 00 | 20 00 |

All original communications designed for publication, works for view, new instruments for notice, exchanges, &c., should be directed the editor, 259 N. Eutaw St., Baltimore, Md.

All advertisements and subscriptions should be addressed to

SNOWDEN & COWMAN,

*Publishers of the Am. Journal of Dental Science.*

86 W. FAYETTE ST. BET. CHARLES & LIBERTY STS.,

BALTIMORE, Md.

